# Mastoid Obliteration After Canal Wall Down Mastoidectomy Using Tissue Engineering Approaches with Polymers, Mesenchymal Stem Cells, and Bioactive Molecules: A Systematic Review

**DOI:** 10.3390/bioengineering13030305

**Published:** 2026-03-05

**Authors:** Kyung Hoon Sun, Cheol Hee Choi, Minseong Kim, Chul Ho Jang

**Affiliations:** 1 Department of Emergency, College of Medicine, Chosun University, Gwangju 61452, Republic of Korea; skhkorea@chosun.ac.kr; 2 Department of Pharmacology, College of Medicine, Chosun University, Gwangju 61452, Republic of Korea; chchoi@chosun.ac.kr; 3 Medical Device Development Center, KBIO HEALTH OSONG Medical Innovation Foundation, 123, Osongsaengmyeong-ro, Cheongju-si 28160, Republic of Korea; mkim@kbiohealth.kr; 4 Department of Otolaryngology, Gwangju Veterans Hospital, Gwangju 62284, Republic of Korea; 5 Department of Otolaryngology, Chonnam National University Medical School, Gwangju 61469, Republic of Korea

**Keywords:** mastoid obliteration, tissue engineering, growth factor, mesenchymal stem cell, polymers, bioprinting

## Abstract

**Background**: Mastoid obliteration following canal wall down mastoidectomy reduces cavity-related morbidity. Conventional obliteration materials act primarily as passive fillers, whereas tissue engineering (TE) strategies aim to achieve biologically active bone regeneration. **Methods**: This systematic review was conducted in accordance with PRISMA 2020 guidelines. PubMed/MEDLINE, Embase, Scopus, and the Cochrane Library were searched from January 2010 to December 2025. Studies evaluating tissue engineering-assisted mastoid obliteration involving growth factors, mesenchymal stem cells, polymer scaffolds, or 3D-printed constructs were included. **Results**: Fifteen studies met inclusion criteria (12 preclinical and three clinical). Polymer-supported MSC constructs demonstrated the most consistent osteogenic enhancement in animal models. Clinical evidence remains limited to small PRP-based case series. **Conclusions**: Preliminary evidence suggests that tissue engineering-assisted mastoid obliteration has regenerative potential, although the evidence is limited by predominantly preclinical data and a moderate-to-high risk of bias. Standardized outcome measures and well-designed prospective clinical studies are required to confirm long-term safety and efficacy.

## 1. Introduction

Canal wall down mastoidectomy remains an effective surgical strategy for advanced chronic otitis media and cholesteatoma. However, the resulting open mastoid cavity may cause persistent otorrhea, debris accumulation, caloric-induced vertigo, and a lifelong need for cavity care [1,2,3,4,5]. Mastoid obliteration has therefore become an integral adjunctive procedure to improve postoperative quality of life [3]. Bioactive ceramics such as hydroxyapatite and bioactive glass are now popular synthetic materials for bone scaffolds in mastoid obliteration. However, they do not meet the requirements for reconstructing the function of the external auditory canal due to their low bioactivity, sluggish in vivo degradation, mismatch between the rate of degradation and the pace of new bone creation, and inadequate support of the external auditory canal wall.

Recent advances in tissue engineering (TE) have introduced biologically active strategies aimed at achieving true bone regeneration through the combination of growth factors, stem cells, polymer-based scaffolds, and advanced fabrication techniques such as three-dimensional (3D) printing [6,7,8,9]. Three essential components are usually involved in tissue engineering techniques: scaffolds, cells, and biochemical and/or mechanical stimulation. Many tactics to encourage tissue development typically start with scaffolds. Polymeric scaffolds are frequently used to promote tissue growth and act as carriers for the transfer of bioactive factors, despite the fact that a variety of scaffold materials are accessible. Among these components, polymer platforms play a pivotal role by providing structural support, facilitating controlled release of bioactive molecules, and enhancing cell viability in irregular mastoid cavities [10,11].

When significant amounts of bone and tissue are lost or destroyed as a result of a traumatic accident or chemical reaction, medical experts and biomedical engineers can now investigate novel therapeutic options thanks to bioprinting technologies [12,13,14]. Due to its potential as a “game-changing” treatment to address the scarcity of organ donors in the US, the capacity to mend bone using bioprinting techniques has drawn particular interest [13].

This systematic review synthesizes the available evidence from 2010 to 2025 on tissue engineering-assisted mastoid obliteration, with a particular focus on the role of polymer-based systems in promoting bone regeneration.

## 2. Materials and Methods

### 2.1. Study Design and Reporting Standards

This systematic review was conducted in accordance with the PRISMA 2020 guidelines.

### 2.2. Eligibility Criteria (PICO Framework)

Population: Human patients or animal models undergoing mastoid obliteration.

Intervention: Mastoid obliteration using tissue engineering approaches, including: growth factors (e.g., BMP-2).

Stem cells (e.g., mesenchymal stem cells), polymer-based scaffolds or carriers, and 3D-printed or architected scaffolds.

Comparator: Conventional obliteration materials or scaffold-only controls.

Outcomes: Primary: Bone regeneration assessed by CT/micro-CT or histology. Secondary: Inflammation, infection, material stability, extrusion, and safety.

Timeframe: Studies published between January 2010 and December 2025.

Study Types: Preclinical animal studies and clinical studies (case series, comparative studies).

Exclusion Criteria: Studies were excluded if they (1) focused solely on middle ear reconstruction without mastoid involvement, (2) used non-biological or strictly inorganic synthetic materials without tissue-engineering components, (3) were case reports with fewer than three subjects, or (4) were review articles, conference abstracts, editorials, or non-peer-reviewed reports.

### 2.3. Information Sources and Search Strategy

Search Strategy: Comprehensive searches were conducted on 5 January 2026. Boolean combinations included: (‘mastoid obliteration’ OR ‘mastoid cavity reconstruction’) AND (‘tissue engineering’ OR ‘mesenchymal stem cell’ OR ‘MSC’ OR ‘BMP-2’ OR ‘growth factor’ OR ‘polymer scaffold’ OR ‘3D printing’ OR ‘bioprinting’).

Inclusion Criteria: Preclinical or clinical studies evaluating tissue engineering-based mastoid obliteration with radiologic or histologic bone regeneration outcomes.

Exclusion Criteria: Narrative reviews, case reports with n < 5, non-English studies, and studies without bone regeneration outcomes.

## 3. Results

### 3.1. Study Selection

**Figure 1:**
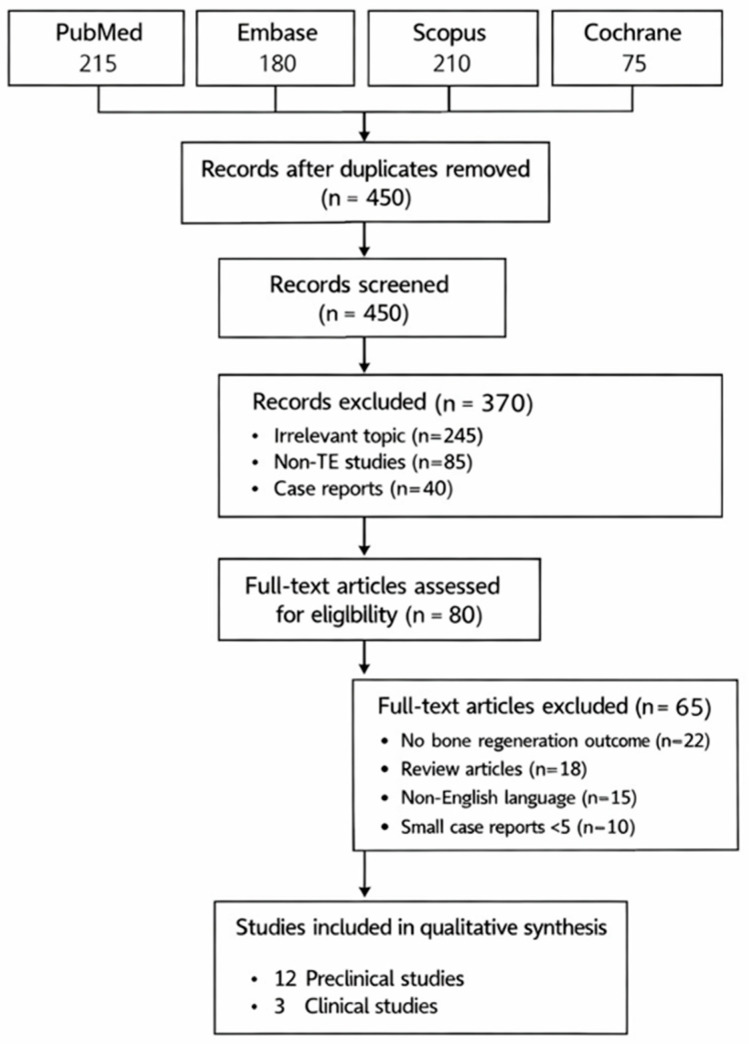
PRISMA 2020 flow diagram illustrating study identification, screening, eligibility, and inclusion for the systematic review.

The study selection process is summarized in the PRISMA 2020 flow diagram (Figure 1). A total of 312 records were identified through database searching. After removal of duplicates, 248 records were screened, of which 61 full-text articles were assessed for eligibility. Ultimately, 15 studies were included in the qualitative synthesis, comprising 12 preclinical animal studies and three clinical studies (Table 1). Overall, the risk of bias across included studies was moderate to high, primarily due to inadequate randomization, lack of blinding, and heterogeneous outcome reporting. These limitations further underscore the need for well-designed prospective clinical trials.

**Figure 2:**
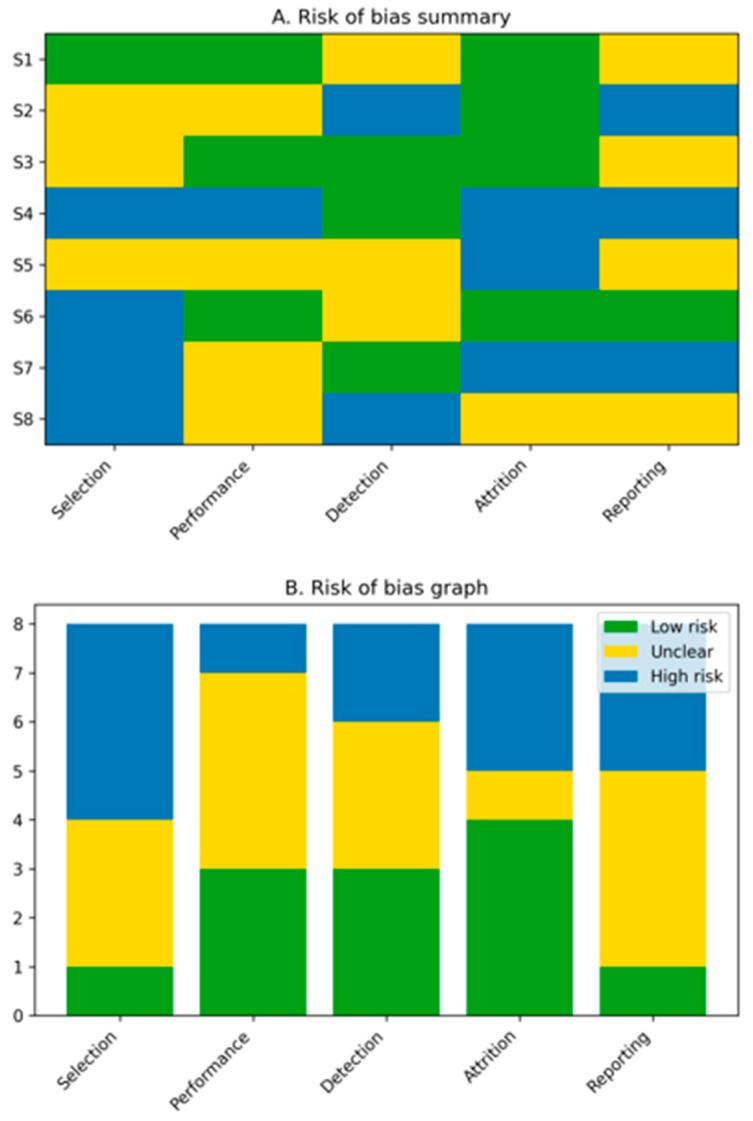
Risk-of-bias assessment of included studies. Preclinical animal studies were evaluated using the SYRCLE risk-of-bias tool, while clinical and translational studies were assessed using the ROBINS-I tool. Overall, the risk of bias was moderate to high, primarily due to limited randomization, lack of blinding, and heterogeneous outcome reporting.

Risk-of-bias assessment revealed an overall moderate to high risk across included studies (Figure 2). Most preclinical investigations demonstrated unclear or high risk of selection and performance bias due to insufficient reporting of randomization and blinding. Clinical studies showed a moderate risk of bias, primarily related to confounding and non-randomized study designs. These limitations highlight the need for standardized methodologies and well-designed prospective clinical trials.

### 3.2. Growth Factor-Augmented Mastoid Obliteration

To date, the only osteoinductive growth factor approved by the Food and Drug Administration (FDA) for use as a bone graft alternative is bone morphogenetic protein-2 (BMP-2). BMP-2 was the most frequently investigated osteoinductive factor in mastoid obliteration models. Several studies demonstrated that rhBMP-2 delivered using polymeric carriers or polymer–ceramic composites significantly enhanced new bone formation compared with scaffold-only controls [5,24,28]. However, a growing and well-documented side effect profile has surfaced as BMP-2’s therapeutic use has increased [29,30,31]. These include incorrect adipogenesis, osteoclast-mediated bone resorption, ectopic bone growth, and surgical inflammation and related side effects. The relative frequency of adverse effects linked to the clinical use of BMP-2, including potentially fatal cervical spine edema, has been validated by a number of extensive investigations. The dose-related adverse effects of BMP-2 highlight the importance of controlled release systems and low-dose strategies. Scaffolds possessing dual characteristics for osteogenesis and angiogenesis exhibit significant potential for bone tissue regeneration [32].

Regenerative medicine has made extensive use of platelet-rich plasma (PRP), a concentration of platelets and other plasma constituents. The release of growth factors that happens during platelet degranulation is the basis for PRP’s regenerative potential. Growth factors that promote cell proliferation and differentiation are released when autologous serum and CaCl2 activate PRP [33,34]. PRP is an autologous biological adjunct widely used in tissue engineering; however, its regenerative effects are transient and nonspecific [35]. It is safe to use and minimally invasive. PRP, which is produced by centrifuging whole blood, contains autologous growth factors such as platelet-derived growth factor (PDGF), insulin-like growth factor (IGF-I), and transforming growth factor (TGF-β), which speeds up the regeneration of platelets, epithelial, endothelial, and epidermoid tissues. Its antinociceptive, anti-inflammatory, and regenerative qualities allow it to be applied directly over the lesion [35]. As a result, it promotes the recovery of chronic injuries. Furthermore, PRP promotes collagen production, angiogenesis, and soft tissue repair. Additionally, it reduces skin scarring and improves the hemostatic response to damage [35].

To date, mastoid obliteration using PRP has been rarely reported [3,15,16]. Askar et al. [15] performed mastoid obliteration using autogenous PRP with cortical bone pate. The clinical study included 21 patients, consisting of 12 females and 9 males. Disease on the left side affected sixteen patients. All procedures were performed uneventfully, with operative times ranging from 90 to 135 min and no intraoperative difficulties reported. Mastoid fistulas and external canal stenosis were not documented at 12 to 16 months of follow-up. In eighteen cases, the tympanic membrane showed good healing. The rebuilt mastoid cavity was smooth and well-aerated, and there were no radiological indicators of recurrence. Although mastoid obliteration using PRP has been rarely reported, PRP has been more commonly used for clinical myringoplasty [36,37,38,39].

It is interesting to note that the liquid components of umbilical cord blood, specifically umbilical cord serum (UCS) and plasma (UCP), represent a major milestone. The primary distinction between these fluids is whether or not they include clotting factors (plasma contains clotting factors, while serum does not). Growth factors, neurotrophic factors, and cytokines with wound healing, anti-inflammatory, anti-aging, and anti-apoptotic qualities are abundant in cord serum [40]. Mastoid obliteration using umbilical cord serum has been performed in preclinical studies [17,18]. Umbilical cord serum has also been used for dry eye disease and other clinical conditions [41].

### 3.3. Cell-Based Approaches

Over the past few decades, mesenchymal stem cells (MSCs) have been considered ideal candidate cells for use as seed cells due to their superior osteogenic competence and strong proliferation potential. However, BMSC extraction is a highly invasive process with minimal cell yield and frequent problems. This encourages scientists to investigate better potential cells such as adipose tissue-derived MSCs [42]. Mesenchymal stem cells (MSCs) derived from bone marrow or adipose tissue were incorporated into mastoid obliteration constructs in multiple studies. MSC-laden polymer scaffolds consistently showed superior osteogenesis compared with MSC injection alone, emphasizing the importance of cell retention and microenvironmental support [19,20]. Additionally, it has been demonstrated that MSCs can stimulate or assist angiogenesis in vitro and produce angiogenic factors and proteases to aid in the development of blood vessels [21]. However, the failure of many bone repair applications might be attributed to the paucity of vasculature alone, which does not provide adequate nutritional supply to the bone graft [32]. By creating a highly biocompatible cell channel, the porous structure of the 3D porous scaffold, which was implanted in a bone defect model, significantly increased the adherence of hADSCs [43,44,45] or vascular cells [26].

Exosomes are extracellular vesicles that range in diameter from 30 to 150 nm. They come from the endosomal system, more precisely as intraluminal vesicles inside multivesicular bodies. They include a range of bioactive substances that can be delivered to target cells, including proteins, lipids, and nucleic acids. By transporting their contents to recipient cells, exosomes facilitate cell communication and can have a variety of biological impacts [22].

Two different co-culture techniques are used to produce spheroids from human umbilical vein endothelial cells (HUVECs) and human bone marrow-derived MSCs. Spheroids of MSCs have become a popular in vitro model for simulating bone formation [46]. Compared to conventional two-dimensional (2D) cultures, the three-dimensional (3D) architecture of spheroids more closely resembles the natural tissue environment. Notably, MSC spheroids have demonstrated great promise in tissue engineering, especially for bone regeneration, and have shown multipotent differentiation potential under suitable environmental signals [26,47].

### 3.4. Polymer-Based Scaffolds and Carriers

Polymer platforms form the structural and biological backbone of most tissue engineering strategies. Polymers play a crucial role in scaffold fabrication because of their advantageous characteristics, including biocompatibility, controlled biodegradability, and suitable mechanical performance. These materials reduce adverse immune reactions, allowing seamless integration with nearby tissues, and their surfaces can be tailored to enhance cell attachment and growth. As polymers degrade gradually in harmony with new tissue formation, they support the repair process without the need for surgical removal once healing is complete [48,49]. Mechanical integrity is particularly important in bone tissue engineering, where scaffolds must tolerate physiological stresses. Through material design, polymers can be tailored to resemble the mechanical behavior of bone, offering an appropriate balance of strength and flexibility to preserve structural stability during regeneration [49]. Furthermore, polymers can be functionalized to deliver bioactive agents, such as growth factors or therapeutic drugs, within the scaffold, thereby improving performance and promoting faster tissue regeneration [50].

Synthetic biodegradable polymers such as polycaprolactone (PCL) [51] and poly(lactic-co-glycolic acid) (PLGA) provide mechanical stability and are particularly suitable for 3D printing applications [52,53]. Natural polymers, including collagen, gelatin, alginate, and fibrin, enhance cell adhesion and facilitate controlled release of growth factors [54,55,56,57,58]. Bone tissue engineering employs polymer-based scaffolds as temporary three-dimensional structures that replicate the natural extracellular matrix and provide support for new bone formation. This strategy integrates cells, bioactive factors, and biomaterials to restore bone defects by fabricating porous scaffolds with adjustable characteristics—such as degradation rate and mechanical stability—using polymers like PLA, PCL, or natural materials such as collagen, thereby promoting osteoblast attachment, growth, and differentiation to facilitate bone regeneration [44,59].

For a polymer scaffold to be effective, it must satisfy strict engineering criteria. Porosity and interconnectivity are essential, with typical porosity levels ranging from 50% to 90% to facilitate cell migration, nutrient diffusion, and blood vessel formation. Biodegradability is also critical, as the scaffold should break down in synchrony with new bone matrix formation, ultimately disappearing without leaving residual foreign material. Mechanical integrity is equally important, as the scaffold must temporarily support physiological loads—particularly in critical-sized bone defects—until the regenerated bone can independently bear stress [60].

### 3.5. 3D-Printed Architected Scaffolds

3D printing technologies enabled precise control over scaffold geometry, porosity, and interconnectivity [61,62,63,64]. In mastoid obliteration models, 3D-printed polymer or polymer–ceramic composite scaffolds demonstrated improved osteo-conduction and spatial stability compared with bulk materials [65]. Researchers have used inkjet printers to successfully create cardiac, brain, and epidermal tissues [66,67]. In terms of bone tissue engineering, 3D-printed scaffolds made of various biomaterials for maxillofacial bone regeneration have shown promise [5,27,68].

The integration of three-dimensional (3D) bioprinting, mesenchymal stem cells (MSCs), and artificial intelligence (AI)-driven design has transformed bone tissue engineering into a predictive and patient-specific discipline. Advanced 3D-printed bone scaffolds provide structural support while replicating the hierarchical porosity and mechanical behavior of native bone, which is particularly critical in complex anatomical regions such as the mastoid [25] and craniofacial skeleton [23].

### 3.6. Critical Analysis of Heterogeneity

The included studies were heterogeneous with respect to animal species (rabbit vs. rat), defect creation and obliteration techniques, scaffold composition (polymer-only vs. composite constructs), bioactive payloads (BMP-2 and other growth factors), and outcome measures (micro-CT parameters, histomorphometry, and inflammation/infection reporting). This variability precluded meaningful quantitative pooling and limited direct cross-study comparisons; therefore, results are presented narratively with emphasis on design features that may explain divergent outcomes.

## 4. Discussion

Most included studies were preclinical and demonstrated enhanced osteogenesis with polymer-MSC constructs. However, heterogeneity in scaffold design, growth factor dosing, and outcome reporting limits cross-study comparisons. Clinical translation remains constrained by regulatory barriers and safety considerations, particularly for BMP-2 and cellular therapies.

### 4.1. Current Status of Mastoid Obliteration

In order to aid in the healing of a mastoidectomy defect, Mosher initially proposed the idea of mastoid obliteration in 1911 [69]. Since that time, numerous reports detailing diverse techniques for mastoid cavity obliteration have been published, significantly advancing the practice of mastoid surgery.

Bioactive materials such as hydroxyapatite and bioactive glass are now the most popular artificial bone repair materials for clinical use in mastoid cavity obliteration. Research has shown that bioactive glass has some flexibility, high biocompatibility, and broad-spectrum antibacterial qualities [70,71,72,73]. However, it does not meet the requirements for reconstructing the function of the external auditory canal due to its low bioactivity, sluggish in vivo degradation, mismatch between the rate of degradation and the pace of new bone creation, and inadequate support of the external auditory canal wall. For more efficient osteogenesis, a tissue engineering approach has been explored. The majority of the studies are limited to preclinical research; BMP-2 and platelet-rich plasma have been tried in clinical mastoid obliteration [1,15,74]. This systematic review highlights a paradigm shift in mastoid obliteration from passive cavity filling toward biologically active bone regeneration using tissue engineering principles [75].

### 4.2. Limitations of Current Tissue Engineering-Based Mastoid Obliteration

Despite growing interest in tissue engineering strategies for mastoid obliteration after canal wall down mastoidectomy, several limitations remain in current clinical practice and published evidence (Table 2). First, most clinical applications are still limited to PRP as the primary biological adjunct [3,15,16]. Although PRP is attractive because of its autologous nature, ease of preparation, and low immunogenic risk, it provides only a short-term and non-specific release of growth factors. Furthermore, inter-individual variability in platelet concentration and preparation protocols results in inconsistent biological activity, limiting reproducibility and standardization across studies.

Second, direct clinical application of MSCs in mastoid obliteration is scarce. While preclinical studies demonstrate that MSCs promote osteogenesis, angiogenesis, and immunomodulation, clinical translation remains constrained by regulatory barriers, ethical concerns, harvesting procedures, and cost. Consequently, high-quality clinical trials evaluating MSC-based mastoid obliteration are lacking. Third, bioactive molecules such as bone morphogenetic proteins (BMPs) and vascular endothelial growth factor (VEGF) have not been widely adopted in otologic reconstruction. Safety concerns related to uncontrolled bone formation, inflammatory reactions, proximity to critical neuro-otologic structures, and unexpected facial swelling have limited their use [76,77]. Moreover, optimal dosing strategies and delivery systems within the mastoid cavity remain undefined. Fourth, heterogeneity in polymer scaffolds and composite biomaterials represents a major limitation. Current studies employ various polymers and polymer–bioceramic composites with differing porosity, degradation rates, and mechanical properties, making direct comparison difficult and precluding robust meta-analysis. Finally, the level of clinical evidence remains low, with most studies consisting of small retrospective case series and short follow-up periods. Long-term endpoints—including durability of obliteration, delayed infection, graft resorption, radiologic evidence of bone maturation, and patient-reported quality-of-life outcomes—are reported inconsistently across studies.

### 4.3. Future Directions: Toward Advanced Regenerative Mastoid Obliteration

Future research should move beyond PRP-centered strategies toward integrated, cell-based, and precision-guided tissue engineering approaches. The use of umbilical cord serum or autologous MSCs represents a promising next step. MSCs derived from bone marrow or adipose tissue offer sustained osteogenic and angiogenic potential and may support long-term bone regeneration while modulating chronic inflammation within the mastoid cavity. To address safety and regulatory concerns, MSC-derived secretomes and extracellular vesicles may serve as effective cell-free alternatives.

In addition, controlled delivery of bioactive molecules is expected to enhance regenerative outcomes [7,8,9]. Advances in polymer chemistry and hydrogel-based carriers may allow localized, sustained release of osteoinductive and angiogenic factors while minimizing adverse effects on adjacent structures such as the facial nerve and inner ear. Three-dimensional bioprinting technologies offer transformative potential for mastoid obliteration [5,28]. Patient-specific scaffolds generated from preoperative CT data could precisely match mastoid anatomy, optimize cavity filling, and enable spatially controlled distribution of cells and bioactive molecules. Ultimately, well-designed prospective clinical trials with standardized outcome measures and long-term follow-up will be essential to validate these advanced tissue engineering approaches and facilitate their translation into routine otologic practice.

## 5. Conclusions

Tissue engineering strategies for mastoid obliteration appear promising for promoting bone regeneration and reducing cavity-related complications in preclinical models. However, the current evidence base is limited by small sample sizes, heterogeneous methodologies, and moderate-to-high risk of bias, with sparse comparative clinical data. These findings should therefore be interpreted cautiously, and well-designed, standardized, long-term clinical trials are needed before routine clinical adoption can be recommended.

## Figures and Tables

**Table 1:** Mastoid obliteration using tissue engineering approaches.

Author	Type	Material	Result	Reference
MESA EIbary et al. [3]	Clinical	PRP only	Enhanced epithelization	Eur Arch Otorhinolaryngol 2025; 282: 6069–6072
Askar SM et al. [15]	Clinical	PRP with bone pate	Achieved complete epithelialization	Ear Nose Throat J 2021; 100: 485–489
MES bd EIbary et al. [16]	Clinical	PRP with bone pate Titanium mesh	Surface of the neocanal wall appeared smooth	Int Arch Otorhinolaryngol 2018; 22: 103–107
Jang CH et al. [17]	Preclinical	Umbilical cord serum PCL/alginate scaffold	GFs in umbilical cord serum enhanced osteogenesis	Int J Pediatr Otorhinolaryngol 2014; 78: 1061–1065
Jang CH et al. [18]	Preclinical	Fibrous collagen, calcium dehiscent HA, umbilical cord serum	Rapid osteogenesis	Int J Biol Macromol 2021; 176: 479–489
Jang CH et al. [19]	Preclinical	MSCs laden PCL/ Collagen scaffold	MSCs enhanced more rapid osteogenesis	RSC Advances 2016; 6: 6259–6265
Choi SW et al. [20]	Preclinical	Tonsil derived MSCs/HA/chitosan patch	MSCs enhanced efficient osteogenesis	ACS Appl Bio Mater 2020; 3: 1008–1017
Skoloudik L et al. [21]	Preclinical	Human MSCs/HA	hMSCs showed a significantly higher ratio of new bone formation	Cell Transplantation 2016; 25: 1405–1414
Park SH et al. [22]	Preclinical	PCL/stromal vascular fraction cells	Autologous SVF cells with PCL are promising	Polymers 2022; 14: 877
Yu F et al. [23]	Preclinical	3D-printed BAG/PCL/BMP-2	Enhanced osteogenesis	Regenerative Therapy 2022; 21: 469–476
Jang CH et al. [5]	Preclinical	3D PCL/alginate/BMP-2	Enhanced osteogenesis	Int J Biol Macromol 2013; 62: 614–622
Jang CH et al. [24]	Preclinical	3D PCL/alginate/BMP-2/UCS	Enhanced osteogenesis	J Industrial Eng Chem 2019; 72: 432–441
Lee J et al. [25]	Preclinical	Highly elastic 3D-printed gelatin/HA/placental extract	GFs from placental extract enhanced osteogenesis	Theranostics 2022; 12: 4051–4066
Kim W et al. [26]	Preclinical	Bioprinted cell constructs with endothelial cell spheroids	Early angiogenesis prominently enhanced	Theranostics 2022; 12: 5404–5417
Jang CH et al. [27]	Preclinical	3D PCL/beta TCP/collagen nanofiber	3D porosity and collagen nanofiber stimulated osteogenesis	Macromol Biosci 2013; 13: 660–668

**Table 2:** Limitations and future directions in tissue engineering-based mastoid obliteration.

Aspect	Current Limitations	Future Directions
Biological adjunct	Predominant use of PRP with short-lived effects	MSCs, MSC-secretome, extracellular vesicles
Growth factor delivery	Non-specific, variable release	Controlled, sustained release of BMPs, VEGF
Scaffold design	Heterogeneous polymers, non-personalized	Patient-specific 3D-bioprinted scaffolds
Regenerative control	Limited spatial and temporal control	Bioprinting with spatial cell/molecule distribution
Personalization	One-size-fits-all approach	AI-driven personalized scaffold optimization
Evidence level	Small retrospective studies	Prospective trials with long-term follow-up

## Data Availability

No new data were created or analyzed in this study.
